# LncRNAs ENST00000499459 and TCONS_00004989 enhance asthma progression in children with house dust mite-induced allergic asthma

**DOI:** 10.1186/s13223-022-00742-7

**Published:** 2022-12-03

**Authors:** Zhang Xude, Feng Shaojie, Guo Beibei, Liu Jingjing, Xu Donghua, Liu Fengxia

**Affiliations:** 1 Department of Allergy, The First Affiliated Hospital of Weifang Medical University/Weifang People’s Hospital, Weifang, People’s Republic of China; 2 The First Clinical Medicine College, Weifang Medical University/Weifang People’s Hospital, Weifang, People’s Republic of China; 3 Department of Rheumatology, The First Affiliated Hospital of Weifang Medical University/Weifang People’s Hospital, Weifang, People’s Republic of China

**Keywords:** LncRNA, Allergic asthma, ENST00000499459, DIXDC1, TCONS_00004989

## Abstract

**Background:**

Long non-coding RNAs (lncRNAs) have been extensively reported to play critical roles in the pathogenesis of various disease, especially in cancer. However, little is known about the role of lncRNAs in the pathogenesis of pediatric allergic asthma.

**Methods:**

High-throughput sequencing analysis was performed to identify differentially expressed mRNAs and lncRNAs in peripheral blood mononuclear cells (PBMCs) from 3 children with allergic asthma and 3 matched healthy controls. Bioinformatics analysis was used to select candidate lncRNAs and mRNAs that may be involved in the pathogenesis of asthma. Candidate lncRNAs were validated in a larger size of asthma patients and healthy controls. Finally, lncRNAs and molecular pathways associated with the pathogenesis of allergic asthma were identified by competing endogenous RNA (ceRNA) analysis.

**Results:**

Five differentially expressed lncRNAs were identified after high-throughput sequencing and verified by real-time PCR. LncRNAs ENST0000631797, TCONS_00004989 and ENST00000499459 were verified to be differentially expressed in allergic asthma. Besides, ENST00000499459/DIXDC1 axis was identified to play a crucial role in allergic asthma after comprehensive ceRNA network analysis.

**Conclusion:**

ENST00000499459 and TCONS_00004989 are potential biomarkers for house dust mite-induced allergic asthma.

## Introduction

### Background

Asthma is a heterogeneous allergic disorder that is usually characterized by airway inflammation, airway remodeling and hyperresponsiveness. It is often accompanied by viral infection or allergen inhalation [1], which seriously affects children’s physiology and quality of life. In the past 30 years, the prevalence of asthma among children aged 0 to14 years has increased in China, from 0.13% in 1990 to 2.12% in 2010 [2]. More than half of asthma cases are allergic asthma induced by house dust mites (HMDs) [3]. Substantial efforts have been made improve the diagnosis, severity assessment and targeted treatment of asthma [4]. Although a number of susceptible asthma-associated gene variants have been identified in a series of genome-wide association studies, the reported risk alleles explain only heritability [5]. The molecular mechanism underlying allergic asthma (AAS) remains unclear. Increasing evidence has shown that the epigenetic modifications modulated by genetic factors and environmental exposures, such as respiratory tract infections and the airway microbiome in early life, are associated with development of AAS [6–8].

In addition to regulating gene expression, lncRNAs also possess transcriptional activation and silencing capabilities, and thus play critical roles in cellular development, differentiation, cell fate, and disease pathogenesis [9]. Previous studies have proven that lncRNAs contribute to the progression of asthma via mediating multiple signaling pathways and acting as regulators of airway inflammation and remodeling [10–12]. Research on the pathogenesis of AAS in children has mostly focused on genetic factors in recent years [13]. However, thus far, there is little research on the role of lncRNAs in the pathogenesis of pediatric AAS.

### Objective

HDM is the most common aeroallergen of children with AAS in China and even worldwide. The aim of this study is to identify novel lncRNAs that might be useful molecules for screening and monitoring HDM-desensitized childhood AAS by a systematic transcriptome analysis of the peripheral blood mononuclear cells from three AAS children and three matched nonatopic subjects.

## Materials and methods

### Patients and healthy controls

Ethics approval for this study was obtained from the Ethics Committee of Weifang People's Hospital. In this study, three AAS patients and three age and sex-matched nonatopic subjects were enrolled for transcriptome analysis of peripheral blood mononuclear cells. All AAS patients were recruited from Weifang People's Hospital, the healthy controls were recruited from the children’s physical examination institution of Weifang. The consent was obtained from participants’ parent/legal guardian. AAS diagnosis was based on asthma exacerbation criteria [14, 15]. All AAS patients had not been treated with medication. Patients with other autoimmune diseases or active/severe infections were excluded from the study. RNA-seq analysis was conducted on samples taken from three AAS patients and three matched healthy controls (Table 1). In subsequent validation, peripheral blood mononuclear cells from 15 AAS patients and 15 matched healthy controls were evaluated (Table 2). Serological examinations, including total immunoglobulin E (tIgE) and Phadiatop (Phadia, Sweden), were performed. The Phadiatop (a mixture of common respiratory allergens containing dust mites) level for the three nonatopic subjects was  ≤ 0.35 PAU/L. In contrast, dust mite allergy was diagnosed when the specific IgE of dust mites was  ≥ 0.35KUA/L.

**Table 1:** The clinical features of patients and healthy controls

Grouping	Serial number	Age (years)	Gender	EOS%	T-IgE (KU/l)	sIgE
Normal control	NC-1	2	female	0.01	17.68	Phadiatop:0.02 PUA/L
	NC-2	7	male	0.3	35.30	Phadiatop:0.01 PUA/L
	NC-3	5	male	0.1	127.00	Phadiatop:0.05 PUA/L
Dust mite sensitized group	T-1	3	male	8.3	222.83	hx2: 13.90 KUA/L
	T-2	4	female	5.4	243.09	hx2:33.53 KUA/L
	T-3	8	male	13.2	143.85	hx2:27.29 KUA/L

*T-IgE* Total IgE, *sIgE* specific IgE, *phad* a mixture of common respiratory allergens containing dust mites. *hx2* house dust mix

**Table 2:** General characteristics of patients (group T) and control group (group NC) participating in the validation study

Group	Gender	Age	T-IgE (KU/l)
NC01	Male	5	0.19
NC02	Female	6	0.02
NC03	Female	3	0.03
NC04	Male	8	0.01
NC05	Female	6	0.02
NC06	Female	3	0.03
NC07	Male	3	0.02
NC08	Male	1	0.11
NC09	Female	5	0.02
NC10	Male	4	0.02
NC11	Male	6	0.02
NC12	Male	6	0.02
NC13	Male	5	0.27
NC14	Male	5	0.06
NC15	Female	4	0.22
T01	Male	8	17.07
T02	Female	4	52.12
T03	Male	5	134.58
T04	Male	8	146.72
T05	Male	8	124.73
T06	Female	3	1.87
T07	Male	9	2.61
T08	Female	9	100
T09	Female	6	200.26
T10	Female	3	9.74
T11	Female	4	100
T12	Male	4	32.65
T13	Female	5	27.07
T14	Male	10	100
T15	Male	9	100

*NC* normal control, *T* test control (AAS patients)

### Sample processing

First, at least 6 ml of peripheral blood from each subject was collected using ethylene diamine tetraacetic acid (EDTA) anticoagulation tubes. Ficol density gradient centrifugation, the most commonly used method to separate peripheral blood mononuclear cells (PBMCs), was performed to separate them from peripheral blood. Total RNAs were extracted with TRIzol (Invitrogen, Carlsbad, CA, USA). Then, total RNAs were extracted according to the instructions of the virvana miRNA Isolation Kit (Ambion), and its quality or integrity was evaluated with Agilent 2100 biological analyzer (Agilent Technology, Santa Clara, California, USA) and expressed with Rin value. Further analysis was performed on samples with RNA integrity number (RIN)  ≥ 7. According to the manufacturer’s instructions, libraries were constructed with TruSeq Stranded Total RNA with Ribo-Zero Gold. The libraries were then sequenced using the Illumina sequencing platform (Illumina NovaSeq 6000), and 150 bp/125 bp paired-end reads were generated. Genes and transcripts exhibiting differential expression were determined based on the log2 (fold-change)*1 criterion and a P value less than 0.05 between two samples.

### Preprocessing and alignment of genomic data

After high-throughput sequencing, the raw reads generated were formatted in FASTQ. Several quality filters were applied to the reads to produce high-quality reads for later analysis. After the adapters were removed using Trimmomatic software, low-quality bases or N-bases were filtered out using a stepwise process. Finally, we were able to acquire clean, high-quality reads with HISAT2, based on which we aligned the reads to the experimental species’ reference genome.

### Analysis of transcript splicing, lncRNA prediction and gene quantification

A binary file containing the results of alignment with the reference genome was generated. To splice the new transcript, the reads were assembled with String Tie software. Then, cuff compare software was used to compare the reference sequence in gene function annotation database to select candidate lncRNA transcripts. Finally, the predicted sequence of the lncRNA was obtained by four protein coding potential prediction software programs, CPC, PLEK, Pfam and CNCI. Bowtie2 and eXpress software with default parameters were used to align the sequencing reading of each sample, known lncRNA sequence and lncRNA prediction sequence, and expression was used for gene quantitative analysis to obtain FPKM value and count value (reading times of each gene in each sample).

### Analysis of co-expression of differential lncRNAs and mRNAs

Before constructing the ceRNA network, the Pearson correlation coefficient analysis was used to estimate the expression correlation between lncRNAs (less than 6000nt in length) and mRNAs. The relationship pairs with correlation coefficients  ≥ 0.8 and P values  ≤ 0.05 were selected. In order to display DE-lncRNAs and gene information more intuitively, we used mapping software to display the same DE-lncRNAs and mRNAs (DE-mRNAs) of the control group in the Circos diagram. When the number of differential lncRNAs was more than 500, the absolute log 2 FC value of the top 500 was taken for analysis.

### Prediction of lncRNA function

Based on the results of differential co-expression, we performed GO analysis of target genes to describe the functional properties of DE-mRNAs and KEGG pathway analysis to investigate the biological pathway of DE-mRNAs. The function of lncRNAs may be obtained in these GO or KEGG enrichment analyses.

### Analysis of lncRNA cis- and trans-acting target genes

Using FEELnc software and based on the data of differential co-expression, we searched all coding genes within 100 k upstream and downstream of DE lncRNAs, as well as the intersection of differential genes with significant co-expression with the lncRNA (Pearson correlation calculation). Similarly, as a result of the co-expression data, the RNA interaction software risearch-2.0 was used to predict the nucleic acid level binding of potential co-expressed lncRNAs and mRNAs. In addition, the screening interaction between lncRNAs and mRNAs can be directly regulated according to the screening conditions and the base binding free energy. Finally, according to the extracted TOP500 (according to the co-expression p value) relationship pairs, we used the network software package to draw the network diagram of lncRNA-mRNA targeting interaction.

### Identification of the DE lncRNAs by fluorescence quantitative PCR

RNA was extracted and reverse transcribed (as described above) in a light cycle using a PerfectStart^TM^ Green qPCR SuperMix kit^®^ 480 II fluorescence quantitative PCR (Roche, Switzerland). System: 2 × PerfectStart^™^ Green qPCR SuperMix, 5 μl; cDNA, 1 μl; 10 μM forward primer, and 10 μM reverse primer, both 0.2 μl; nuclease-free H_2_O, 3.6 μl. PCR procedure: 94 °C for 30 s, 94 °C for 5 s and 60 °C for 30 s, 45 cycles. At the end of the cycle, the specificity of the product was detected in the form of melting curve. The specific method was as follows: the temperature increased slowly from 60 ℃ to 97 ℃, and 5 times per ℃ of the fluorescence signal were collected. The 2^−∆∆CT^ method was used to calculate RNA expression.

### Statistical analysis

Screening of the differentially expressed mRNA and lncRNA was conducted by the whole transcriptome sequencing. By default, FC2 and p < 0.05 were used as differential screening criteria. The p-values were analyzed using the variance analysis software DESeq 2. DESeq2 was implemented as a package for the R statistical environment and running in the linux system. During differential analysis, p and q are generally output at the same time [Benjamini & Hochberg check p value]. Upon the CT value was obtained by qPCR, the expression of the quantitative gene was calculated by 2^-ΔΔCt. The difference was calculated compared to the basal level of the gene expression, and the p value and FC value were calculated using the R package. In general, the p-value is used for difference calculation by default.

## Results

### DE- lncRNA and DE-mRNA profiles

**Fig. 1:**
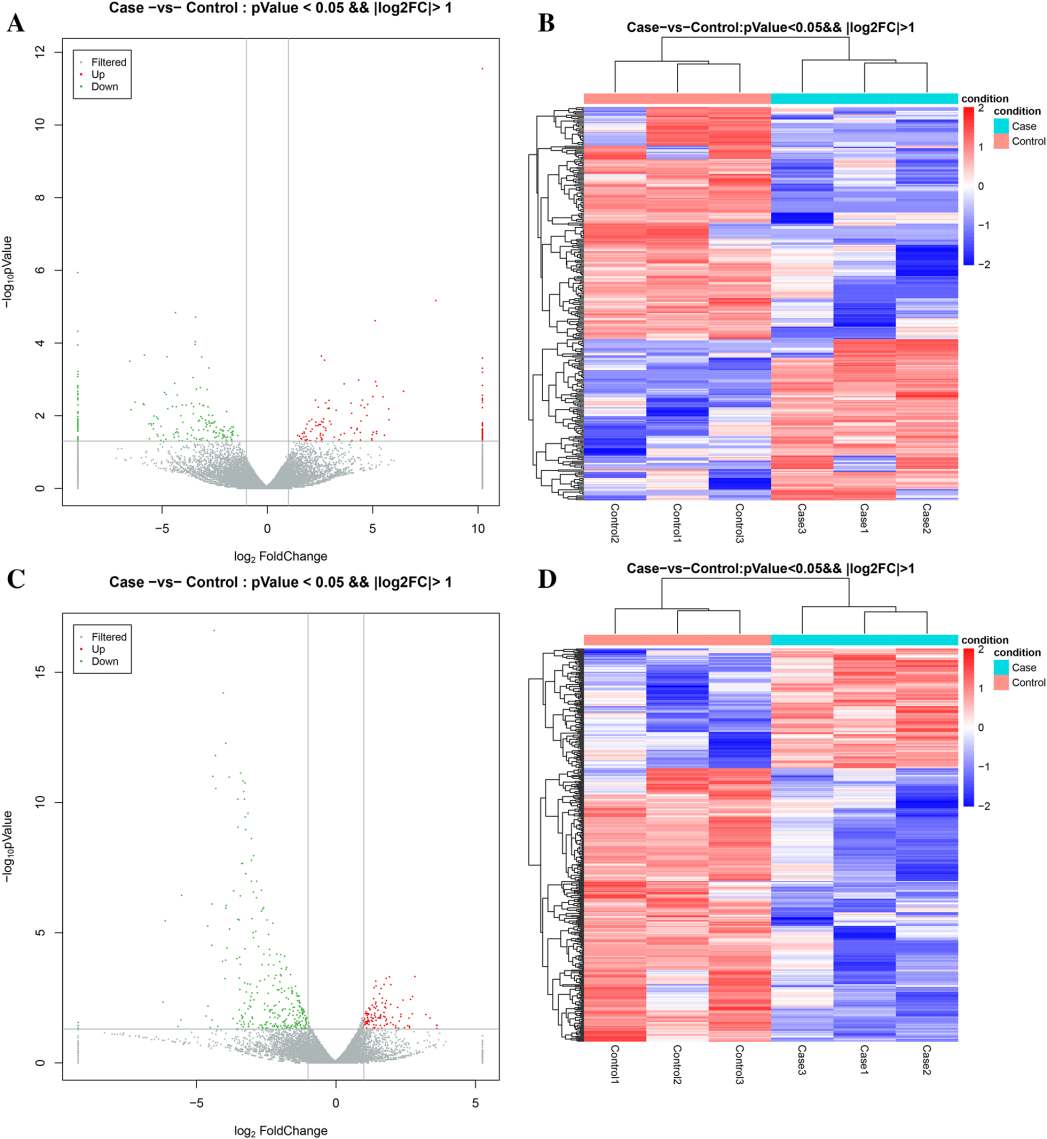
The expression profile of LncRNAs and mRNA in PBMCs of children with allergic asthma and healthy individuals. Volcano plots (**A**) and hierarchical clustering analysis (**B**) of DE-lncRNAs, volcano plots (**C**) and hierarchical clustering analysis (**D**) of DE-mRNAs. Different color in volcano plots represent up (red), and down-regulated (green) genes, respectively. Colors of red and blue in Hierarchical clustering analysis represent up/down regulated genes with p-value  < 0.05 and changes larger than twofold, respectively

To investigate the expression levels of lncRNAs in patients with house dust mite-induced AAS and healthy controls, PBMCs of the two groups were analyzed using lncRNA and mRNA microarrays. The volcano plot and hierarchical clustering analysis (Fig. 1A, B) revealed the abnormally expressed lncRNAs in patients with AAS compared with healthy controls. A total of 318 lncRNAs were aberrantly expressed among 51,540 tested lncRNAs, including 130 upregulated and 188 downregulated lncRNAs. Top 10 upregulated and downregulated lncRNAs were listed in Table 3. In this study, 404 out of 20,030 tested mRNAs showed abnormal expression, including 123 upregulated mRNAs and 281 downregulated mRNAs. (Table 4). The volcano plot and hierarchical clustering analysis (Fig. 1C, D) showed the aberrantly expressed mRNAs of AAS patients.

**Table 3:** LncRNAs differentially up/down regulated in PBMCs of children with AAS and healthy individuals (top 10 each)

lncRNA_symbol	lncRNA_id	log2 fold change	P value	Expression (up/down)
THAP7-AS1	NR_027051.1	3.30	0.007	Up
XLOC_009310	NR_027051.1	2.71	0.012	Up
AC008966.1	ENST00000499459	2.14	0.013	Up
AP000787.1	ENST00000543573	5.81	0.013	Up
EBLN3P	ENST00000630598	2.02	0.014	Up
XLOC_009438	TCONS_00021148	4.47	0.014	Up
LOC105370108	XR_001749777.1	2.99	0.014	Up
LOC105376467	XR_930775.2	2.60	0.014	Up
RAB30-AS1	ENST00000533528	2.33	0.015	Up
LOC105369197	XR_001753990.1	2.68	0.015	Up
AP000547.3	ENST00000592918	− 4.33	< 0.001	Down
XLOC_002298	TCONS_00004989	− 3.37	< 0.001	Down
AP001189.1	ENST00000447519	− 3.39	< 0.001	Down
LINC00989	ENST00000508174	− 3.40	< 0.001	Down
XLOC_011093	TCONS_00024816	− 3.08	< 0.001	Down
XLOC_011093	TCONS_00024817	− 2.74	< 0.001	Down
LOC112267861	XR_002956941.1	− 3.49	< 0.001	Down
LOC102724808	XR_924835.2	− 3.15	0.002	Down
AF127936.2	ENST00000455253	− 3.34	0.002	Down
LINC01002	ENST00000631797	− 2.83	0.002	Down

**Table 4:** mRNAs differentially up/down-regulated in PBMC of children with AAS and healthy individuals (top 10 each)

Gene_ID	log2 fold change	P value	Expression (up/down)
MYL9	− 4.34	< 0.001	Down
SPARC	− 4.02	< 0.001	Down
ABLIM3	− 3.93	< 0.001	Down
CMTM5	− 4.30	< 0.001	Down
CAVIN2	− 3.40	< 0.001	Down
EGF	− 4.39	< 0.001	Down
GP9	− 3.80	< 0.001	Down
PPBP	− 3.31	< 0.001	Down
TUBB1	− 3.24	< 0.001	Down
SEC14L5	− 4.28	< 0.001	Down
TENM4	2.85	< 0.001	Up
SUSD4	1.94	< 0.001	Up
LRFN2	1.83	< 0.001	Up
NEO1	1.45	< 0.001	Up
ITGA11	1.75	0.001	Up
VWA5B2	1.99	0.001	Up
GCNT4	1.46	0.001	Up
SLC4A10	1.39	0.001	Up
ACE	1.65	0.001	Up
SCG3	1.59	0.002	Up

### Analysis of coexpression of DE-lncRNAs and DE-mRNAs

**Fig. 2:**
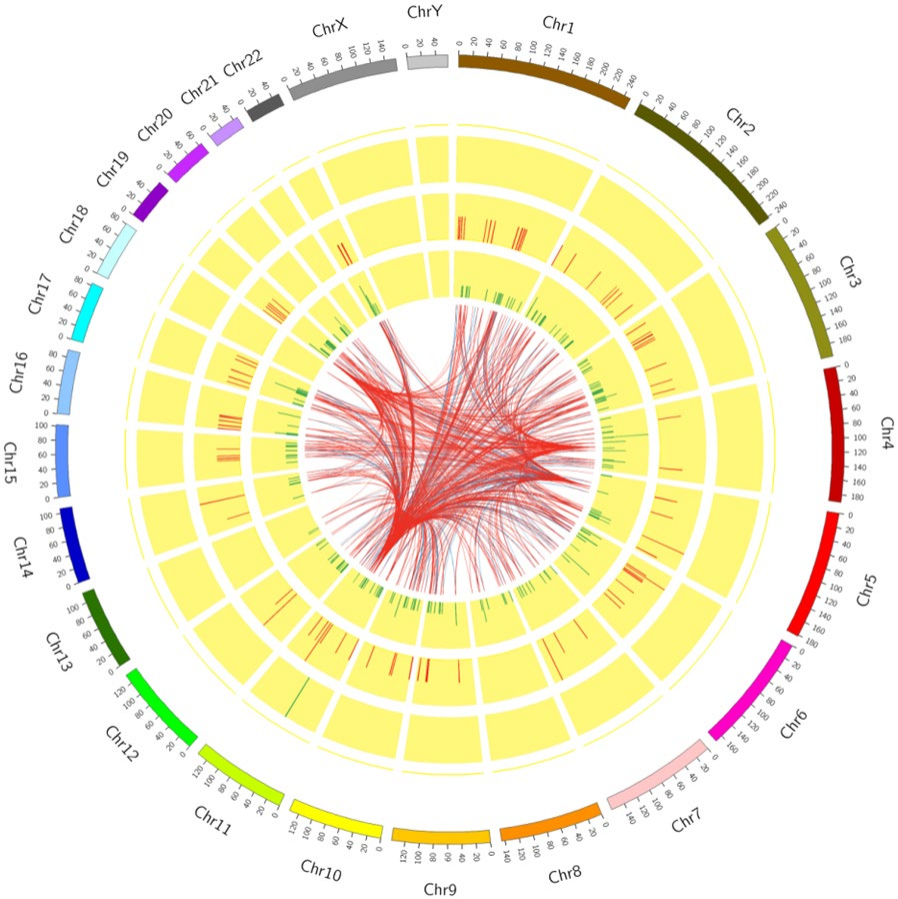
The outer circle represents autosomal distribution; second and third circles represent differential expression on chromosomes. The red lines indicate upregulation while green lines indicate downregulation. The number of differentially expressed genes in this region is expressed by the height of the column. The distribution of DE lncRNAs on chromosomes in the fourth and fifth circles was the same as that of genes; The inner line shows the corresponding relationship between LncRNAs and gene coexpressed by TOP500

A total of 129 upregulated and 188 downregulated lncRNAs and 123 upregulated and 281 downregulated mRNAs were identified in AAS children. Tables 3, 4 showed top 10 aberrantly expressed lncRNAs and mRNAs in AAS, respectively. For a more intuitive display of the DE-lncRNAs and genes, the DE lncRNAs and mRNAs of the comparison group were shown in a Circosdiagram (Fig. 2).

### LncRNA cis-regulation and trans-regulation of target genes

**Fig. 3:**
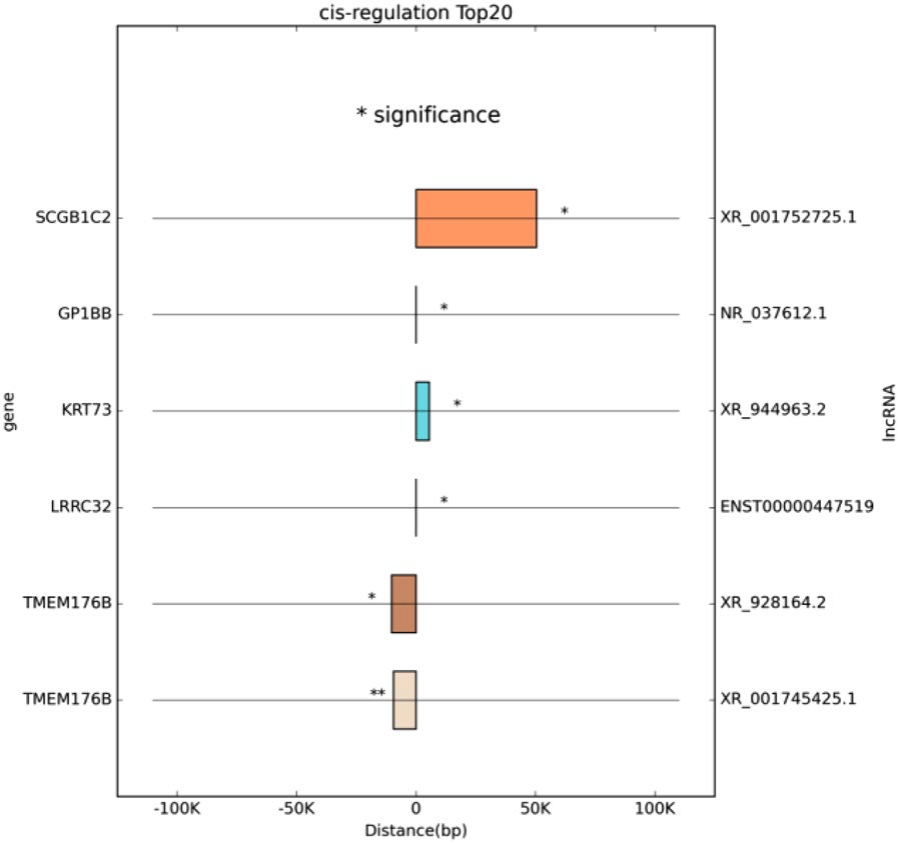
Top 20 lncRNAs’ cis-regulation of targeting gene. (*:p < 0.05, **:p < 0.01)

**Fig. 4:**
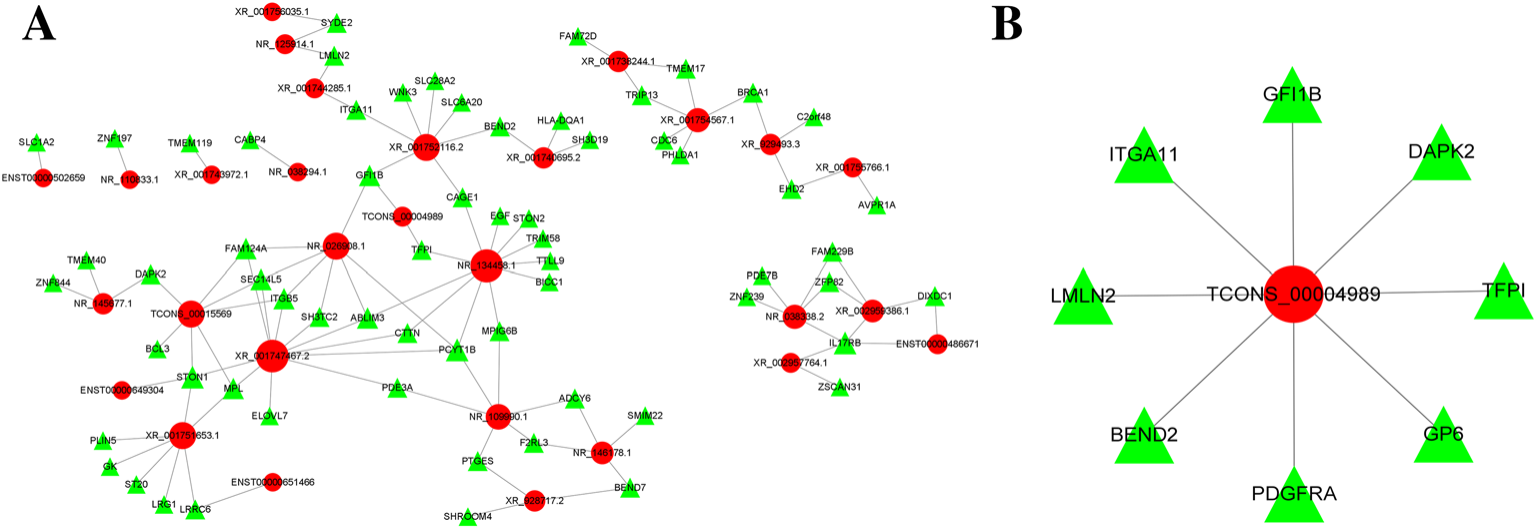
Trans-regulation network of lncRNAs and corresponding genes in this study (**A**) and detailed TCONS_ 00004989 trans regulated genes (**B**)

Top 20 lncRNAs’ cis-regulation of targeting gene were screened out, ENST00000447519 vs LRRC32 was one of six pairs of genes with statistical significance in cis-regulation (Fig. 3). Moreover, trans-regulation network of lncRNAs and corresponding genes in this study was also shown, TCONS_ 00004989 trans regulated genes were analyzed in details (Fig. 4A, B).

### Identification of AAS-related lncRNAs using a basic mRNA–miRNA–lncRNA network

**Fig. 5:**
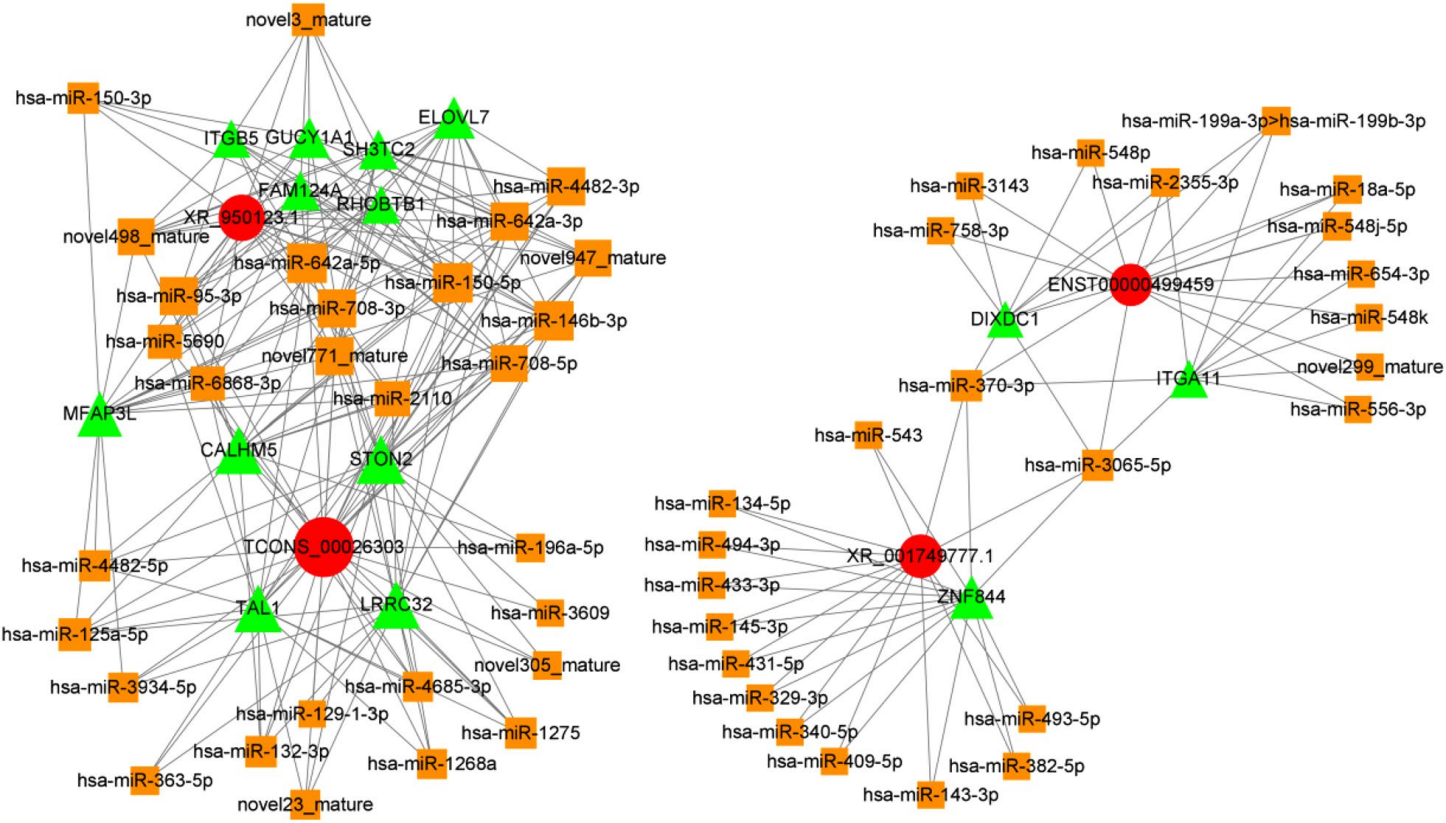
ceRNA_mRNA-miRNA-lncRNA network

The lncRNAs targeted by DE-miRNAs were predicted by using LncBase. We conducted lncRNA screening according to the expression amount (fpkm) and difference (meeting the following conditions at the same time): (1) Padj  < 0.1 or P value  < 0.01; (2) at least one group with an average fpkm  > 3; (3) at least one group with an average fpkm  < 50; and (4) FC  > 2 or FC  < 0.5. We identified 5 lncRNAs as AAS-related molecules: downregulated TCONS_00004989, upregulated ENST00000447519, downregulated TCONS_00024816, upregulated ENST00000499459, and downregulated ENST00000631797. Subsequently, DE-lncRNAs in the PBMC-DE-miRNA-DE-mRNA network was built, and 40 miRNAs were included (Fig. 5).

### Validation of the predicted DE-lncRNAs in AAS children

**Fig. 6:**
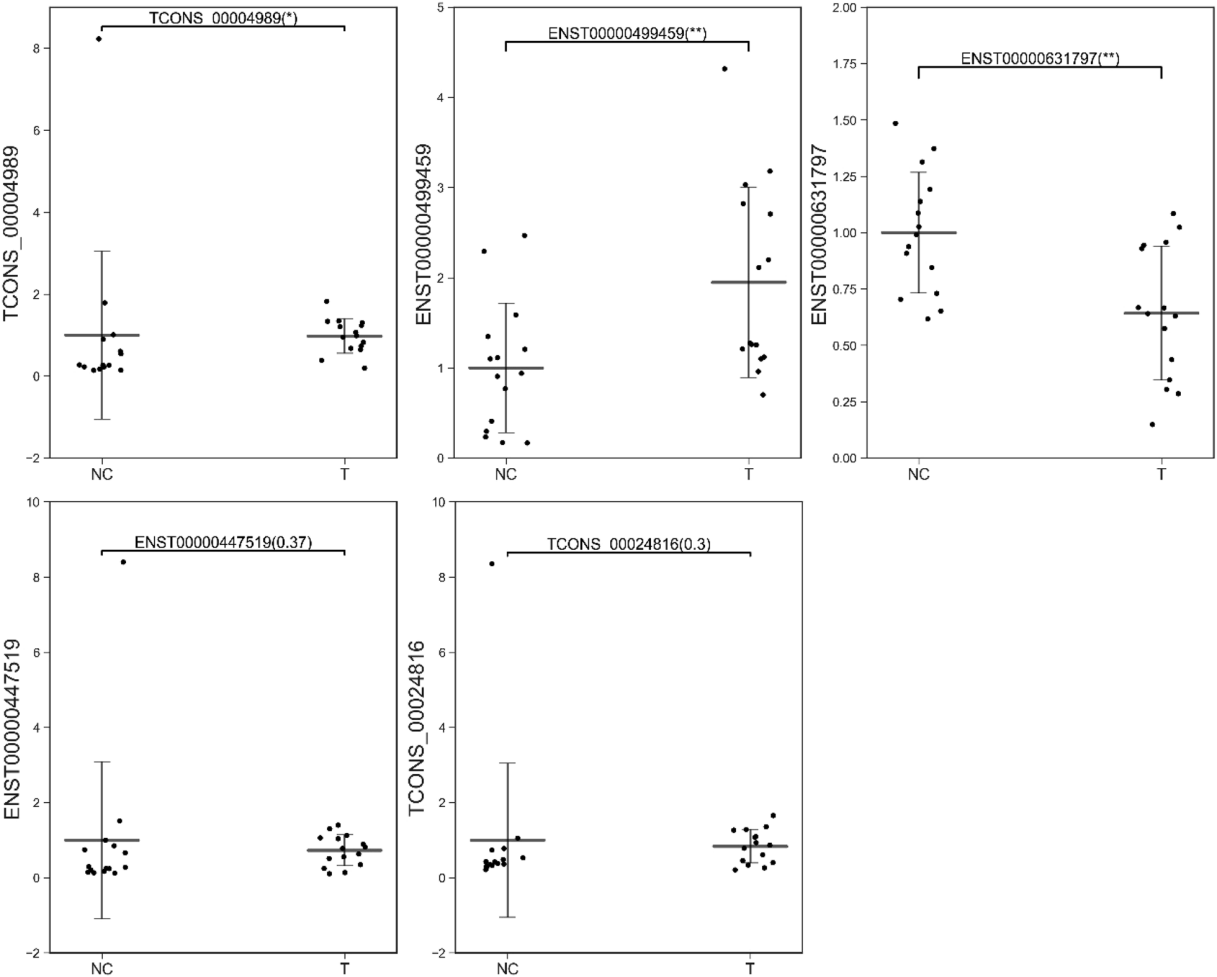
2-△△ CT of gene real-time quantitative PCR value of five validated DE-lncRNAs from 15AAS patients and 15 healthy controls(*:p < 0.05, **:p < 0.01). *NC* normal control group, *T* test group

Among the DE-lncRNAs in the microarray analysis, five DE-lncRNAs were validated by real-time PCR in the PBMC samples from 15 AAS patients and 15 healthy controls (Table 2). Among the five lncRNAs, two were found to be differentially expressed in PBMCs from AAS patients, namely, upregulated ENST00000499459 and downregulated ENST00000631797 (Fig. 6).

### GO and KEGG enrichment analyses of aberrantly expressed lncRNAs

**Fig. 7:**
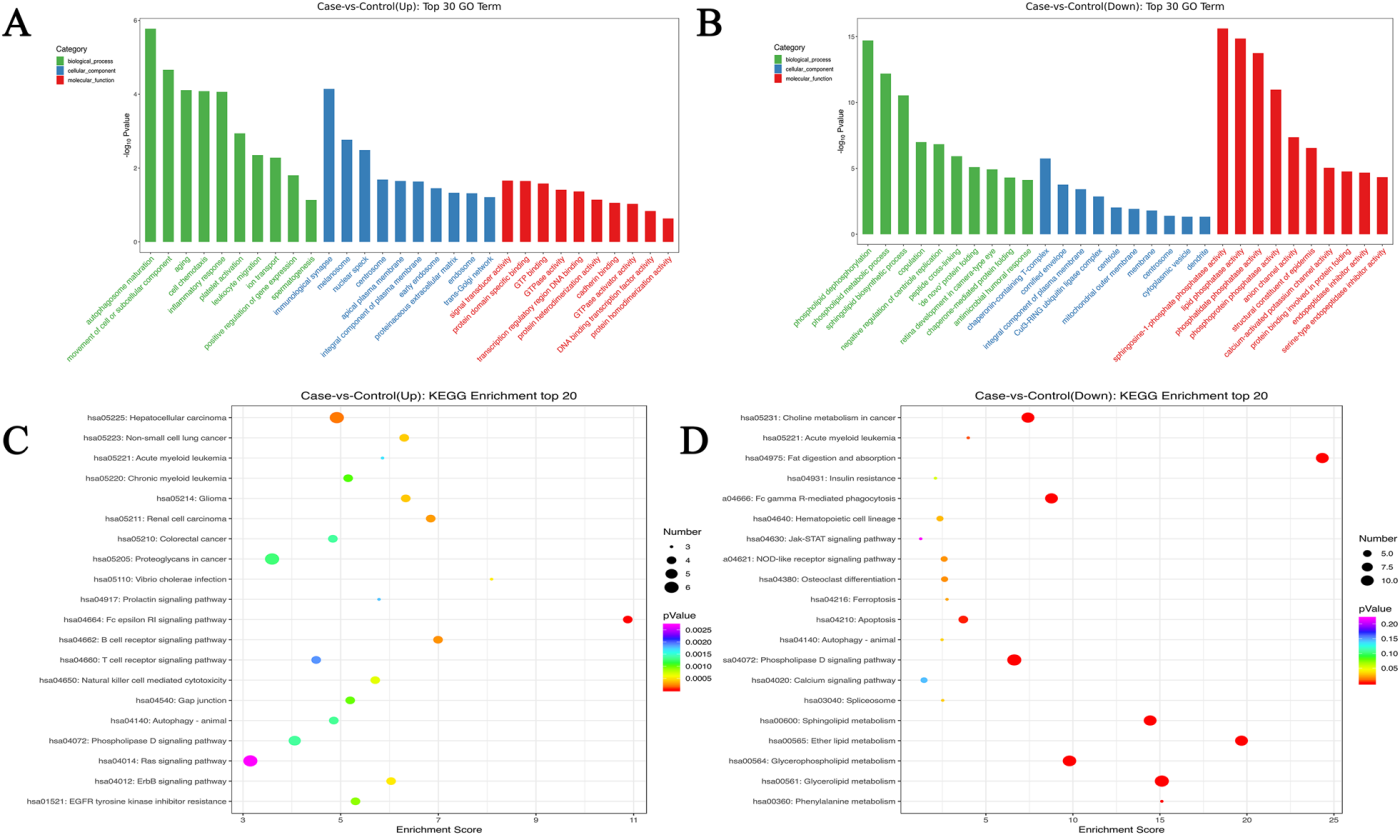
Analysis of GO and KEGG enrichment for all aberrantly expressed lncRNAs. **A** GO enrichment analysis for strongly upregulated expressed lncRNAs in AAS (top 10). **B** GO enrichment analysis for significantly downregulated expressed lncRNAs in AAS (top 10). **C** KEEN enrichment analysis for significantly upregulated expressed lncRNAs in AAS (top 20). In the figure, the Y axis corresponds to KEEN entries, the X axis corresponds to enrichment score, and the shapes identify KEEN path IDs and KEEN terms. The size of points corresponds to the number of differential genes in KEEN entries; moreover, the colored bubbles change from purple, blue, green, to red. The smaller the enrichment p value is, the greater its significance. **D** KEEN enrichment analysis for significantly downregulated expressed lncRNAs in AAS (top 20)

Based on the differential co-expression results, a hypergeometric distribution algorithm was used to analyze GO and KEGG enrichment [16]. These GO or KEGG enrichment analyses of lncRNAs might be closely related to their functions. In the study, DE lncRNAs were significantly enriched for 543 GO-biological process (GO-BP) terms. The most strongly upregulated genes were associated with biological processes, inflammatory response, autophagosome maturation, movement of cell or subcellular component, aging, and cell chemotaxis. The results also showed lncRNA enrichment in 78 GO-cellular component (GO-CC) terms, such as immunological synapse (a markedly upregulated term), chaperonin-containing T-complex, Cul3-RING ubiquitin ligase complex, and 137 GO-molecular function (GO-MF) terms, such as signal transducer activity protein domain specific binding, sphingosine − 1 − phosphate phosphatase activity, and phosphoprotein phosphatase activity. The top 10 GO-BP/CC/MF terms are displayed in Fig. 7A, B. For KEGG analysis, we found 53 significantly enriched KEGG pathways, including hsa04014: Ras signaling pathway, hsa04660: T cell receptor signaling pathway, hsa04664: Fc epsilon RI signaling pathway that were strongly upregulated, and significantly downregulated hsa04020: Calcium signaling pathway, hsa04630: Jak − STAT signaling pathway, and hsa04666: Fc gamma R − mediated phagocytosis (Fig. 7C, D).

## Discussion

Allergic asthma, a reversible airway inflammatory disease caused by aeroallergen sensitization, is the most common type of asthma. The onset of allergic asthma is most often in childhood, and the most frequently detected allergen in childhood worldwide is dust mites. Many previous studies on cells (eosinophils, macrophages, neutrophils), cytokines and chemokines have revealed some aspects of the pathogenesis of allergic asthma. In recent years, the role of lncRNAs in regulating the disease process of allergic asthma and acting as therapeutic targets has drawn more and more attention [17]. Furthermore, lncRNAs affect immune cell function, signal transduction and cytokine secretion by preventing mRNA degradation, which leads to aggravation of asthma.

There is increasing evidence that the lncRNA–miRNA–mRNA axis is involved in asthma pathogenesis, for example, MEG3 and MALAT1 [18–20] are involved. However, research on lncRNAs in the pathogenesis of AAS, a typical Th2-associated inflammatory disease, is rather limited.

To explore the underlying molecular mechanisms of AAS, we integrated ceRNA network analysis with large-scale high-throughput sequencing data. In this study, we synthesized the p value of DE-lncRNAs and lncRNA–miRNA interactions and screened five lncRNA molecules that might play an important role in the pathogenesis of AAS. Among them, TCONS_00004989 binds its upstream gene GLYAT, which plays a major role in the process of monocarboxylic acid metabolism and benzoyl-CoA metabolism, by antisense. However, there have been no reports on the pathogenesis of the GLYAT gene in asthma and other diseases. TCONS_00004989 also competitively inhibits the expression of the tissue factor pathway inhibitor (TFPI) gene through trans regulation, which is considered to be a molecular marker of elevated chronic bronchial inflammation and remodeling in allergic asthma [21]. LRRC32, formerly known as GAPR, is the cis regulatory target gene of lncRNA-ENST00000447519, which was also identified as a key regulator of negative activated T-cell proliferation and a negative regulator of cytokine secretion [22, 23]. Further clinical studies have found that the C11orf30-LRRC32 region impacts tIgE levels and asthma onset in asthmatic Chinese children. [24, 25]. In this study, lncRNA-ENST00000447519 was significantly upregulated in the systematic transcriptome analysis, and our experimental findings coincided with the above research results. Another lncRNA, TCONS_00024816, which was downregulated in asthmatic patients in this study, targeted the gene CXXC4, an inhibitor of the canonical wnt pathway required for the formation of anterior neural structures. Thus far, relevant research on the mechanism of asthma has been conducted. Possibly due to the limited sample size, the above-mentioned three lncRNAs did not show significant differences between patients and healthy controls in subsequent validation.

Differential expression of TCONS_00004989, lncRNAENST0000631797 and ENST00000499459 was validated by real-time PCR in PBMC samples from 15 AAS patients and 15 matched healthy controls. The regulatory target mRNAs of ENST00000499459 were DIXDC1 and ITGA11 in the mRNA-miRNA-lncRNA ceRNA network. To our knowledge, there are still no reports on the correlation between these mRNAs and asthma or other allergic diseases. Therefore, we believe that ENST00000499459 is a newly discovered lncRNA molecule related to the disease risk, severity, and inflammatory state of dust mite-mediated AAS.

It is well known that DIXDC1 is a positive regulator of the Wnt signaling pathway [26], and recent molecular research on the DXIDC1/Wnt axis is concentrated in the field of tumorigenesis [27, 28]. The association between the Wnt signaling gene and asthma susceptibility was previously demonstrated by genome-wide association study [29]. Moreover, Wnt-3a expression directly affects human mast cells to produce the chemokines IL-8 and CCL8 and can further promote the development of allergic asthma [30, 31]. In this study, we demonstrated that the increased expression of lncRNA ENST00000499459 and its positively associated dxidc1 gene in children with asthma caused by mite sensitization activated the Wnt signaling pathway, which eventually led to the onset of asthma. Thus, future work will be required to validate the underlying ENST00000499459/DXIDC1/Wnt signaling pathway in allergic asthma. In this study, another gene, ITGA11, co-expressed with ENST00000499459, has attracted much attention because of its increased expression in cancer-associated fibroblasts (CAFs) leading to the progression of tumors, especially non-small cell lung cancer [32, 33]. Nonetheless, there is no evidence for its role in Th2 inflammation, particularly asthma. ITGA11, as a cell surface adhesion receptor, mediates the adhesion between cells and the extracellular matrix [33]. We speculate that the increased expression of ITGA11 in PBMCs would indicate the inflammatory state of asthma exacerbation to some extent.

However, there remain several points to be improved in this study. First, the detailed mechanism by which lncRNA ENST00000499459 interacted with DXIDC1/ITGA11 in allergic asthma was not investigated, which requires further functional experiments. Second, in the validation experiment, the sample size was relatively limited. Some potential asthma-related DE-lncRNAs should be further assessed in future studies with large sample size.

In conclusion, lncRNAs ENST00000499459 and TCONS_00004989 are promising biomarkers for predicting the exacerbation, severity, and inflammation assessment of dust mite-induced AAS. These findings are preliminary, which warrant to be investigated further in the future.

## Data Availability

Data in this study are available from the corresponding authors upon request.
